# Light environments affect herbivory patterns but not reproductive performance of a multivoltine specialist moth, *Pareuchaetes pseudoinsulata*

**DOI:** 10.1038/s41598-020-74079-9

**Published:** 2020-10-09

**Authors:** Osariyekemwen O. Uyi

**Affiliations:** 1 Department of Animal and Environmental Biology, University of Benin, P. M. B. 1154, Benin City, Nigeria; 2 Department of Zoology and Entomology, University of Fort Hare, Private Bag X1314, Alice, 5700 South Africa; 3 Present Address: Department of Entomology, Pennsylvania State University, University Park, PA 16802 USA

**Keywords:** Ecology, Zoology, Ecology

## Abstract

Unravelling the responses of insect herbivores to light-environment-mediated variation in the traits of their host plants is central to our understanding of the nutritional ecology of, and factors driving the population dynamics in, these species. This study examined the effect of light environment (shaded vs full-sun habitat) on leaf toughness and leaf nutritional quality in *Chromolaena odorata* (an invasive species in West Africa) and related these attributes to the abundance, herbivory patterns and reproductive performance of a multivoltine specialist moth, *Pareuchaetes pseudoinsulata* (a biological control agent). In this system, plants growing in shaded areas in the field experienced more herbivory and had higher herbivore abundance than those growing in full-sun. In the laboratory, *P. pseudoinsulata* larvae consumed significantly greater amounts of shaded foliage relative to full-sun foliage. However, reproductive performance metrics such as mating success, pre-oviposition period, number of eggs laid, duration of egg laying, egg hatchability, and adult longevity in *P. pseudoinsulata* did not differ according to foliage types. Reduced leaf toughness, increased water and nitrogen contents in shaded leaves coincided with increased leaf consumption by the larvae of *P. pseudoinsulata*. In summary, this study showed for the first time that light environments affect herbivory patterns but not reproductive performance of *P. pseudoinsulata* and hypothesized that high foliar nitrogen and water contents in shaded leaves resulted in feedback and necessity consumption patterns.

## Introduction

Conspecific plant species vary their leaf chemistry and phenotypic characteristics when grown in different habitats^1–4^. Herbivorous insects, in turn, are affected by these differences, with complex variations in their performance in response to leaf properties^5–9^. A burgeoning plethora of evidences suggest that phytochemical and phenotypic variation in individuals of the same plant species may be modulated by sunlight intensity in their environments^7,8,10,11^.

The carbon-nutrient balance (CNB) hypothesis^12–14^ postulates that leaves of plants growing under low light levels (e.g. in shaded habitat) should contain relatively more mineral nutrients, especially nitrogen and relatively less carbon-based secondary compounds (e.g. phenolics, tannins) compared to plants growing in full-sun environment. According to the CNB hypothesis, leaves of plants growing in shaded environments should have lower carbohydrate content^1,7^, reduced tannins and phenolics^3,15^, increased foliar nitrogen^3,8^ and higher water content^11,15^. Leaf toughness is also reduced in plants growing under shade^1,11^. These trait differences have been hypothesized to cause increased insect abundance, increased herbivory and improved insect performance on shaded leaves^12–14^.

Studies on responses of insect herbivores to light-mediated changes in the leaf characteristics of their host plant often show mixed correspondence with some of the studies unequivocally supporting the CNB hypothesis and others producing neutral, or at times contradictory results^3–6,8–10^. Many previous studies on light-environment effects on herbivore–host plant interactions have observed increased leaf consumption and herbivore abundance in shade environments^1^ or increased insect herbivore performance on leaves from shaded habitats^8,9,11^. In contrast, some studies have observed higher herbivore abundance and greater leaf consumption in full-sun environments^2,3^ and better insect herbivore performance on full-sun leaves^3^. Therefore, the specific manner in which insect herbivores respond to sunlight-mediated changes in their host plants remains open to debate. From an evolutionary perspective, plants growing in full-sun habitats develop some morphological, physiological, and/or biochemical adaptations resulting in plants with tough leaves and low water content (an adaptation for desiccation resistance), high defense compounds (e.g. tannins and phenolics: an adaptation to prevent or limit herbivory), and low foliar nitrogen relative to shaded plants^1,3,11,15^. These sunlight-induced adaptations in the leaves of full-sun plants may influence plant and herbivore water balance and/or alter nutrient intake by herbivorous insects^7,8^. This alteration over time may lead to varying coevolutionary dynamics that can later influence the ability of herbivores to utilize foliage of plants, in different light environments (e.g. shade vs full-sun habitat) or with different photosynthetic pathways (e.g. C3 vs C4 plants; C3: soft leaves, lower carbon content and higher foliar nitrogen; C4: tougher leaves, higher carbon content, lower foliar nitrogen)^16–19^. For example, females of *Bicyclus safitza* (Westwood) (Lepidoptera: Nymphalidae) laid more eggs and larval performed better on C4 shade grasses than on either C4 grasses from open habitats or C3 grasses, however, hatchling survival was equally good on C4 or C3 shade grasses^19^. These findings^19^ suggest that some herbivores may have adapted to utilise hardened C4 grasses, which may also give them competitive advantage over C3 grasses that prefer shade environments.

Our current knowledge of the nutritional ecology of the specialist herbivore, *Pareuchaetes pseudoinsulata* Rego Barros (Lepidoptera: Erebidae: Arctiinae) (commonly known as Chromolaena leaf feeding moth) which was introduced into Nigeria for the biological control of *Chromolaena odorata* (L.) King & Robinson (commonly known as Siam weed), is preliminary^9^. *Chromolaena odorata* (a plant with classical C3 photosynthetic pathway) is an invasive alien shrub native to the Americas (from southern USA to northern Argentina) that was introduced into Nigeria in the late 1930s^20,21^. It spread to other countries in Africa causing a serious threat to agriculture, biodiversity conservation and livelihoods^20,21^. Studies which integrate variability in leaf traits (due to light intensity) and insect abundance and performance are key in advancing our understanding of the interaction between *P. pseudoinsulata* and its host. In an earlier study, Uyi et al.^9^ examined the effects of light-mediated changes on the preference and performance of *P. pseudoinsulata* on leaves obtained from shaded and full-sun habitats. The study demonstrated prolonged development time in *P. pseudoinsulata* larvae that fed on full-sun foliage, but survival, growth rate and host suitability index of the moth did not significantly differ between full-sun and shaded foliage. The earlier study^9^ relied on laboratory data to show preference and performance of *P. pseudoinsulata* and did not document the effects of light-mediated changes in the leaf characteristics of *C. odorata* plants on leaf consumption and key herbivore reproductive performance metrics. Therefore, this current study conducted both field and laboratory studies to address these knowledge gaps by asking the following questions: (1) do larvae of *P. pseudoinsulata* prefer to feed on shaded or full-sun foliage in the field? (2) is leaf consumption by *P. pseudoinsulata* greater on shaded or full-sun leaves? (3) does feeding on foliage from varying habitats (shaded versus full-sun) influence the reproductive performance of the herbivore? And (4) do light environments influence *C. odorata* leaf nutritional quality and leaf toughness? To answer these questions, several leaf characteristics of *C. odorata* plants growing under shaded or full-sun conditions were measured in the laboratory, whereas the insect abundance, herbivory patterns and reproductive performance of *P. pseudoinsulata* were evaluated on *C. odorata* foliage from shaded or full-sun habitats in both field and laboratory conditions.

## Materials and methods

### Study organisms

Although *C. odorata* grows in a wide range of vegetation types, it is known to be intolerant of deep shade, but grows well in semi-shaded or full-sun conditions^22,23^. Because the damage caused by *C. odorata* was economically and ecologically too significant to ignore, a specialist, multivoltine (6–8 generations per year) leaf feeding moth, *P. pseudoinsulata* was introduced into Nigeria as a biological control agent in the early 1970s^21^. This dull yellow moth lays its eggs in batches on the undersides of the leaves of *C. odorata*; the eggs hatch after an average of 5 days and development from neonate larvae to adults typically takes between 30 and 45 days^24^. Other aspects of the biology and ecology of this nocturnal moth are documented in Cruttwell^25^ and Muniappan et al.^24^. Although *P. pseudoinsulata* was released in Ibadan, southern Nigeria, in the 1970s, it was thought not to have established probably due to predation by ants^26^. In 2009, the moth was discovered in locations near Benin City, in southern Nigeria and was thought to have spread into Nigeria from Ghana (where the moth established in the mid-1990s) or established incognito following the initial release efforts in Nigeria (see discussion in^27^). Anecdotal and empirical reports suggest that the leaf feeding activities of the moth, significantly decreased the density of *C. odorata* plants from a cover of 80% down to 30% in Ghana and Guam^28,29^. Despite its rarity in the field in Nigeria, occasional recovery and outbreaks of the moths are sometimes evident during the wet/rainy season. These outbreaks occur on isolated and dense *C. odorata* plants growing in either open or shaded habitat. There are no reports on the preference and performance of this moth in the field, hence our study seeks to address this gap.

### Abundance and feeding activity of *Pareuchaetes pseudoinsulata*

The study was carried out in an abandoned farmland with extensive *C. odorata* infestation in Uson village near (6° 14′ 30.77″ N, 6° 04′ 41.37″ E) Benin City, in southern Nigeria. The field chosen consisted of full-sun (or open) and shaded habitats with *C. odorata* plants of between 0.5 and 3 m in height. This land was initially used as a farmland for growing cassava, okra and maize. The *C. odorata* plants growing in the shaded environment had a straggling habit with broader leaves while those growing in full-sun were firm and more upright. The full-sun habitat was fully exposed to sunlight and was dominated by *C*. *odorata*, with a sparse population of *Aspilia africana* Pers. (C.D. Adams) (Asteraceae), while the shaded habitat was partially exposed to sunlight and consisted *C. odorata*, oil palm trees, *Elaeis guineensis* Jacq (Arecaceae), cashew trees, *Anacardium occidentale* L. (Anacardiaceae), plantain trees, *Musa* species (Musaceae) and a sparse population of *A. africana*. Light intensity was measured at 1 m above ground level by a LX-135 light meter, Lutron, Taipei, Taiwan. The probe was held near the main stem with a few branches and many leaves. The light intensity measurements were taken between 08h30 and 09h30 on a partly cloudy day on June 14, 2015 during the intense rainy season. Light intensity differed significantly between the two habitats (mean ± SE = 2 109.50 ± 21.18 vs. 431.17 ± 5.09 lx, for full-sun and shaded environment, respectively; GLM ANOVA: F_1,19_ = 374.28, P = 0.0001).

The locals permitted the use of the land and agreed to leave the land fallow for a one-year period during which the experiment was conducted. Prior to this study, an earlier report^27^ confirmed the presence of *P. pseudoinulata* in Uson village. To decipher the preference of and damage caused by *P. pseudoinsulata* under shaded and full-sun environment, a total of eight 1 m^2^ quadrats along a 40 m transect were each laid out in both habitat types on every sampling occasion. Four transects were sampled per habitat type. The distances between each transect was 20 m. The number of *P. pseudoinsulata* larvae (any instar) on *C. odorata* plants growing in both habitats was assessed by means of visual counting upon careful examination of plants in the quadrats. Leaf damage caused by *P. pseudoinsulata* was examined in each quadrat, based on the number of leaves defoliated. Herbivory was recorded by assigning each quadrat to one of the following scores: no defoliation (zero leaf damage = 0), light defoliation (less than 25% total leaf damage = 1), medium defoliation (50% leaf damage = 2), high defoliation (75% leaf damage = 3) and complete defoliation (100% leaf damage = 4). All four transects were sampled monthly from March to December 2015.

### Origin and maintenance of insect culture for laboratory study

The larvae used in this study were obtained from eggs laid by adult females in the laboratory of the Animal and Environmental Biology Department, University of Benin, Benin City, Nigeria. The original parents were collected as late instar larvae in February 2015 at an abandoned farmland in Evbuabogun village (6° 15′ N, 5° 38′ E) near Benin City, Nigeria, where the insect was discovered in 2009^27^. Following the successful emergence of *F*_1_ adults, two males and two females were placed in aerated 700-ml plastic containers, each with a 5-cm-diameter mesh window at the top, with *C. odorata* stem cuttings plugged into moistened cotton-wool wrapped with aluminium foil for egg laying. They were provided with a cotton-wool ball soaked with a 50% (wt/vol) honey solution and were kept in the Animal and Environmental Biology Departmental Laboratory. Hatched larvae (from eggs laid by females) were fed on cuttings with fully expanded leaves obtained from plants growing in semi-shaded environments. The resulting adults (one virgin female and two newly eclosed males) were placed in 700-ml containers as described above. Offspring (first instar larvae) from eggs laid by *F*_2_ females were used for these studies. All experiments were conducted in the Department of Animal and Environmental Biology Laboratory. Temperatures ranged from 23.27 to 29.13 °C during the rearing and experimental periods (mean ± SE: 25.17 ± 2.1). This temperature range is similar to the range that the moth is likely to encounter in field situations.

### Leaf area consumption

Total (i.e. lifetime) leaf consumption by larvae of *P. pseudoinsulata* reared on leaves from two different microhabitats (full-sun vs shaded habitat within the vicinity of the University of Benin Teaching Hospital, Benin City [6° 39′ N, 5° 56′ E], Nigeria) was measured on individual larvae provided with equivalent amounts of food. The full-sun habitat was fully exposed to sunlight and was dominated by *C. odorata* plants, with a sparse population of *Mimosa diplotricha* C. Wright ex Sauvalle (Mimosaceae), whereas the shaded habitat was partially exposed to sunlight and consisted of plantain trees, *Musa* species (Musaceae). Light intensity was measured at 1 m above ground level by a LX-135 light meter, Lutron, Taipei, Taiwan. The probe was held near the main stem with a few branches and many leaves. The light intensity measurements were taken between 08h30 and 09h30 on a partly cloudy day on June 15, 2015 during the intense rainy season. Light intensity differed significantly between the two habitats (mean ± SE = 1 949.50 ± 23.46 vs. 342.10 ± 7.65 lx, for full-sun and shaded environment, respectively; GLM ANOVA: F_1,19_ = 4244.96, P = 0.0001). Field investigation revealed that *P. pseudoinsulata* was absent from this site.

Larvae were raised individually from day of hatching to pupation in 100-ml aerated plastic containers with a circular net screen window (2.5 cm diameter on top for ventilation) lined at the bottom with moistened filter paper to maintain relative humidity. The larvae were fed on one of the two *C. odorata* foliage types for the duration of their development. This protocol presented at least three main advantages; (a) feeding larvae in isolation prevented biases due to competition and consequent food deprivation, (b) accurate quantification of the leaf area consumed by a single individual from first instar to pupation could be made, and (c) variations due to microhabitat effects (full-sun vs shade) could be accounted for. Larvae (n = 50 per foliage type) were fed with fresh, fully expanded leaf tissues (taken from the upper half of the plants) every 24 h and their frass was removed at the same time interval for hygienic reasons^27^. All leaf materials were obtained fresh from over eight plants per habitat on each collection date. The daily use of new leaf tissues is consistent with field observations of *Pareuchaetes* species preferentially feeding on undamaged leaves in the presence of an abundant food supply. Although the use of excised leaves in the determination of insect survival and performance has been a subject of debate^30,31^, a recent study found that egg and larval survival did not differ between leaves in intact plants and excised leaves in the specialist herbivore, *Pieris napi* (L.) (Lepidoptera: Pieridae, Pierini), whereas larval growth was slightly, but significantly, faster on leaf-cuttings^32^. The use of excised leaves is a standard method for providing uniform materials in the laboratory feeding studies of this kind^33^. All containers were placed in a tray inside a transparent plastic bag (600 × 450 mm) to prevent desiccation.

The area of leaf tissue consumed per individual larva per day was assessed by scanning images of the leaf tissues before and after feeding, with a digital scanner (HP Deskjet F380, San Diego, USA). Scanned images were analyzed using COMPU Eye Leaf and Symptom Area program developed by Bakr^34^ (available at https://www.ehabsoft.com/CompuEye/LeafSArea/). The above procedure was continued each day for each larva until feeding ceased in the last instars. The sexes of the resulting pupae were determined. Female pupae were weighed as an index of the adult body size. Upon completion of the experiment, 25 and 24 females successfully eclosed from the shaded and full-sun diet, respectively. Therefore, only leaf consumption and pupal mass data from female individuals that survived to eclosion were considered for statistical analysis. All eclosed adult males were not considered for analysis. Larval survival, total development time and growth rate of *P. pseudoinsulata* were not considered in this study—as these have been previously reported elsewhere^9^. The study was conducted in the wet (rainy) season.

### Reproductive performance

When the adults from the leaf consumption trial eclosed, one virgin female and two newly eclosed males from the matching diet treatment were placed in 700-ml containers as described above (as was done for oviposition of field-collected adults) but they were provided with stem cuttings (with leaves) of the plant type (shaded or full-sun) they had fed on as larvae. Adult females that fed on shaded leaves were placed in the same container with males that fed on the same diet as larvae (from the above experiment and from the laboratory culture), while adult females that fed on full-sun leaves were presented with males that fed on the same food type. Twenty-four (24) replicates each were used for both the full-sun and shaded trials. The containers were provided with a cotton-wool ball soaked with a 50% honey solution. The containers and leaves were examined daily to record (i) pre-oviposition period, (ii) mating success (percentage of matings), assessed by production of fertile eggs, (iii) numbers of eggs laid, (iv) duration of egg laying and (v) adult female longevity. Percentage eggs that hatched (egg hatchability) was also recorded. Although the rearing conditions were relatively homogeneous, the quality of the foliage offered to the larvae could not be regarded as uniform because it was obtained fresh from different light environments. Therefore, any noticeable change in herbivore fitness could be attributed to light environment effects on leaf quality (shaded vs. full-sun habitat).

### Measurement of leaf characteristics

Specific leaf weight (SLW) (which provides a physiological estimate of ‘leaf toughness’^35^) of 100 fully expanded leaves (taken from the upper half of the plants) obtained from 20 plants (five leaves per plant) in June 2015 was estimated in each habitat following the methods described in Steinbauer^35^. A hole punch (diameter 5.54 mm) was used to take a leaf disc from the middle of the leaf. Fresh discs were weighed before being wrapped in individual pieces of aluminium foil and were oven dried at 64 ºC for 72 h before being reweighed. Specific leaf weight was calculated for each habitat using the formula: SLW = dry weight of leaf disc (mg)/area of hole punch in mm^2^. In the same month, leaf materials were also collected from 10 randomly selected *C. odorata* plants along a 20-m transect in each habitat and were subjected to analyses at the Soil Science Laboratory, University of Benin, Benin City, Nigeria. The leaves were dried for 72 h at 65 °C and nitrogen and carbon contents were determined as a percentage of dry weight using a TRUSPEC CN analyser (LECO, Michigan, USA). After ashing of a subsample, phosphorus content was determined colorimetrically^36^. The amount of the total non-structural carbohydrate (NSC) in leaves was analysed using the acid hydrolysis procedure^37^. Finally, water content (%) was calculated using the formula: [(leaf fresh weight – leaf dry weight)/leaf fresh weight] × 100%.

### Statistical analysis

Larval abundance and feeding damage score in all quadrats per habitat were pooled and the differences in these parameters between shade and full-sun habitats were analysed using Mann–Whitney U-test because the data violated the assumptions of normality of data and homoscedasticity of variance (after Shapiro–Wilk’s and Bartlett’s tests). Total leaf consumption by larvae reared on shaded versus full-sun foliage was evaluated using General Linear Model Analysis of Variance (GLM ANOVA). The effect of leaves from the two habitats (shaded and full-sun) on pupal mass, longevity and number of eggs was evaluated using GLM ANOVA. The effect of larval diet (full-sun and shade foliage) on pre-oviposition period and duration of egg laying was analysed using a t-test, while percentage egg hatchability and mating success were evaluated using Mann Whitney U-test and Pearson’s χ^2^ test. Differences in leaf characteristics between shaded and full-sun habitats were compared using a one-way analysis of variance. Except for the GLM ANOVA that were performed using IBM SPSS Statistical software version 20.0 (SPSS, Chicago, IL, USA), all other analyses were performed using GENSTAT 12.0 (VSN International, Hemel Hempstead, UK).

## Results

### Larval abundance and feeding damage

**Figure 1:**
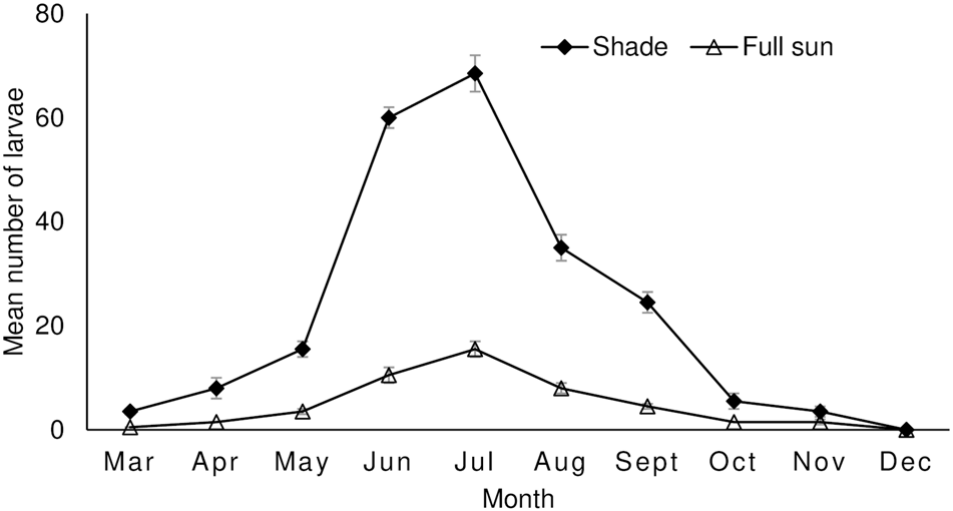
Temporal distribution (mean ± SE) of *Pareuchaetes pseudoinsulata* per m^2^ in shaded and full-sun habitats.

**Figure 2:**
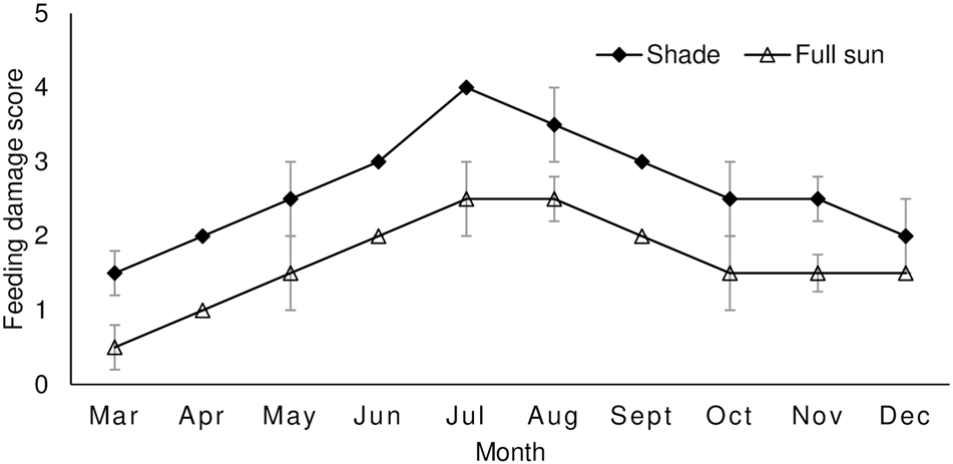
Monthly feeding damage score (mean ± SE) on leaves of *Chromolaena odorata* caused by *Pareuchaetes pseudoinsulata* in shaded and full-sun habitats. Means of feeding damage scores without visible error bars indicate standard error values of zero or close to zero.

*Pareuchaetes pseudoinsulata* larvae were more abundant in shaded habitat compared to full-sun habitat (Table 1). Feeding damage on the leaves was significantly higher on plants growing under shaded conditions than on those plants growing in full-sun conditions (Table 1). Larval abundance and feeding damage scores on leaves in both habitats showed a steady monthly increase until a peak was reached in July before a steady decline (Figs. 1 and 2).

**Table 1:** *Pareuchaetes pseudoinsulata* larval abundance and feeding damage score (± SE) (sample sizes are in parentheses) caused by *P. pseudoinsulata* larvae on *Chromolaena odorata* growing in shaded and full-sun habitats.

Parameter	Shade	Full-sun	Test	P-value
Abundance	22.15 ± 3.72 (40)	8.85 ± 1.46 (40)	U = 532.0	< 0.001
Damage rating	2.62 ± 0.11 (40)	1.60 ± 0.13 (40)	U = 334.0	< 0.001

### Leaf consumption

**Figure 3:**
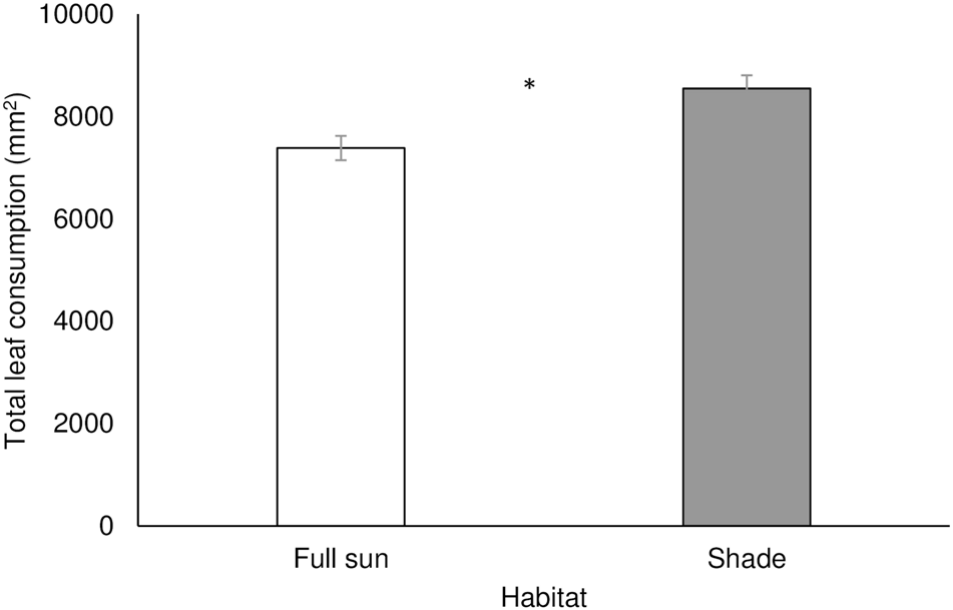
Mean (± SE) leaf consumption of *Pareuchaetes pseudoinsulata* reared on *Chromolaena odorata* leaves from two habitat conditions (full-sun vs. shade). The asterisk indicates a statistical difference (GLM ANOVA: P < 0.05).

Total leaf area consumed by individuals of *P. pseudoinsulata* varied significantly according to habitat type (F_1,48_ = 11.37; P = 0.002) (Fig. 3). Larvae that were reared on shaded foliage consumed 15.76% more leaf area than their counterparts that fed on full-sun foliage.

### Reproductive performance

**Figure 4:**
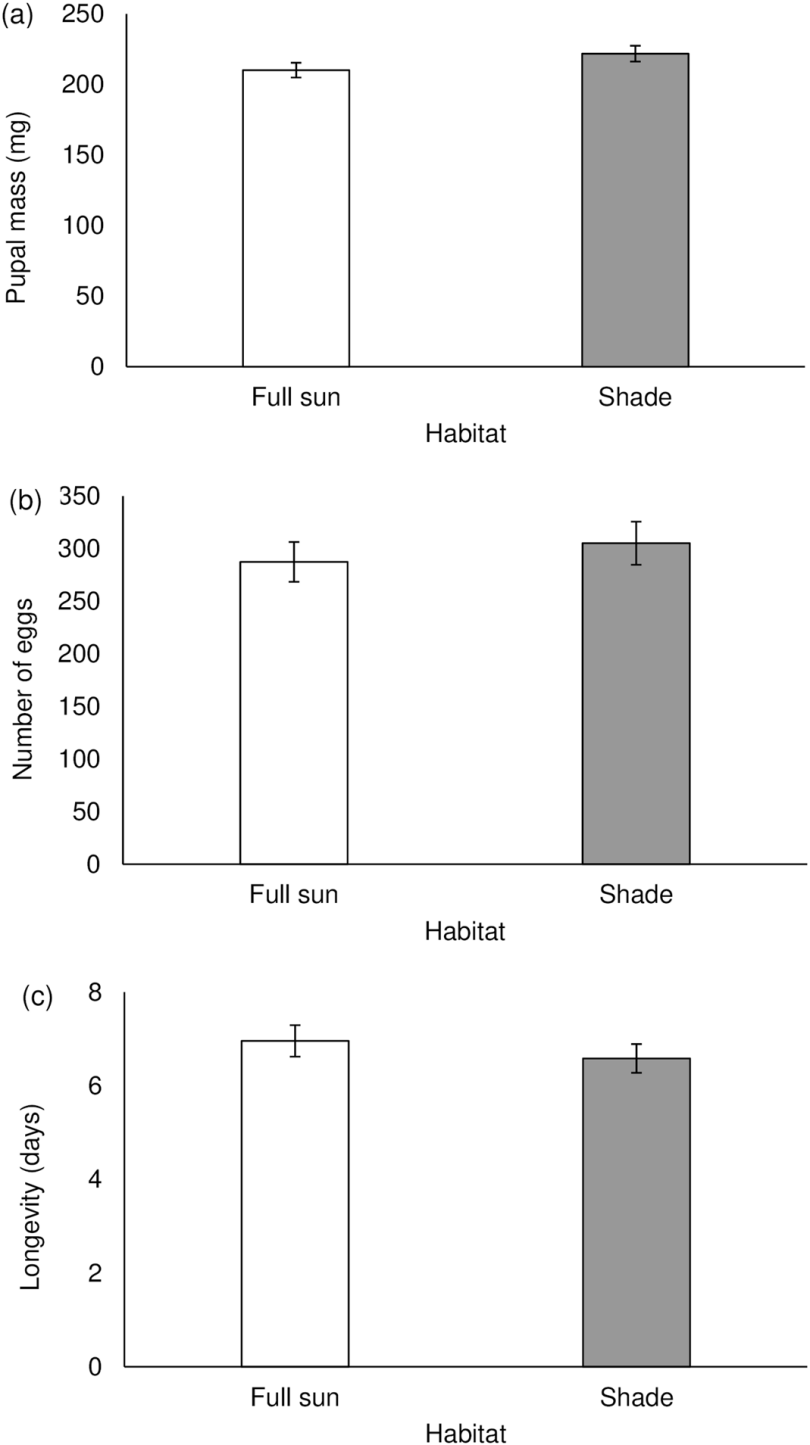
Mean (± SE) Pupal mass (**a**) number of eggs (**b**) and adult female longevity (**c**) of *Pareuchaetes pseudoinsulata* reared on *Chromolaena odorata* leaves from two habitat conditions (full-sun vs. shade).

Female pupal mass (F_1,48_ = 2.33; P = 0.133), number of eggs (F_1,47_ = 0.41; P = 0.526) and female longevity (F_1,47_ = 0.56; P = 0.458) did not vary as a function of foliage type (Fig. 4a–c). Similarly, other herbivore reproductive metrics such as mating success, duration of egg laying, pre-oviposition period, and egg hatchability did not significantly differ between shaded and full-sun foliage (Table 2).

**Table 2:** Some reproductive parameters of *Pareuchaetes pseudoinsulata* (mean ± SE) reared on *Chromolaena odorata* leaves from shaded or full-sun habitats.

Parameter	Full sun	Shade	Test	*P*
Mating success (%)	72.112 ± 3.364 (24)	96.712 ± 3.141 (24)	*χ*^*2*^_1_ = 0.827	0.410
Egg hatchability (%)	93.269 ± 3.169 (3617)^a^	94.230 ± 3.470 (3893)^a^	*U* = 647.3	0.092
Duration of egg laying (days)	4.924 ± 0.513 (24)	4.515 ± 0.425 (24)	*t*_*1,45*_ = 1.31	0.081
Pre-oviposition period (days)	1.516 ± 0.160 (24)	1.572 ± 0.103 (24)	*t*_*1,45*_ = − 0.74	0.51

The numbers in parentheses represent the sample size.

^a^Eggs were from 15 adult females of *P. pseudoinsulata.*

### Correlations between leaf consumption and herbivore performance

**Figure 5:**
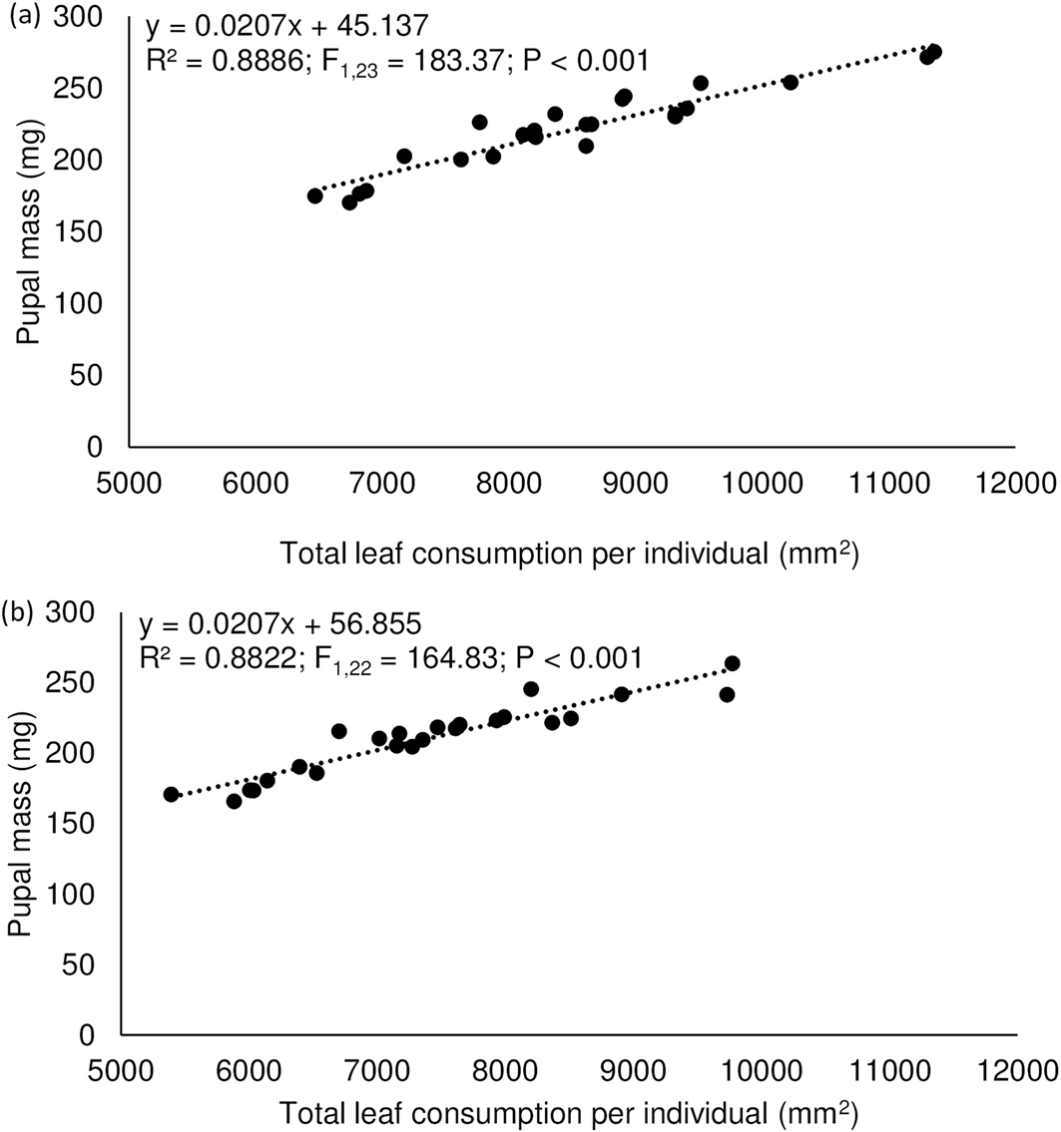
Relationship between female pupal mass (mg) and leaf consumption (mg) by the larvae of *Pareuchaetes pseudoinsulata* fed on shaded (**a**) and full-sun (**b**) leaves. Feeding was from first instar until pupation.

**Figure 6:**
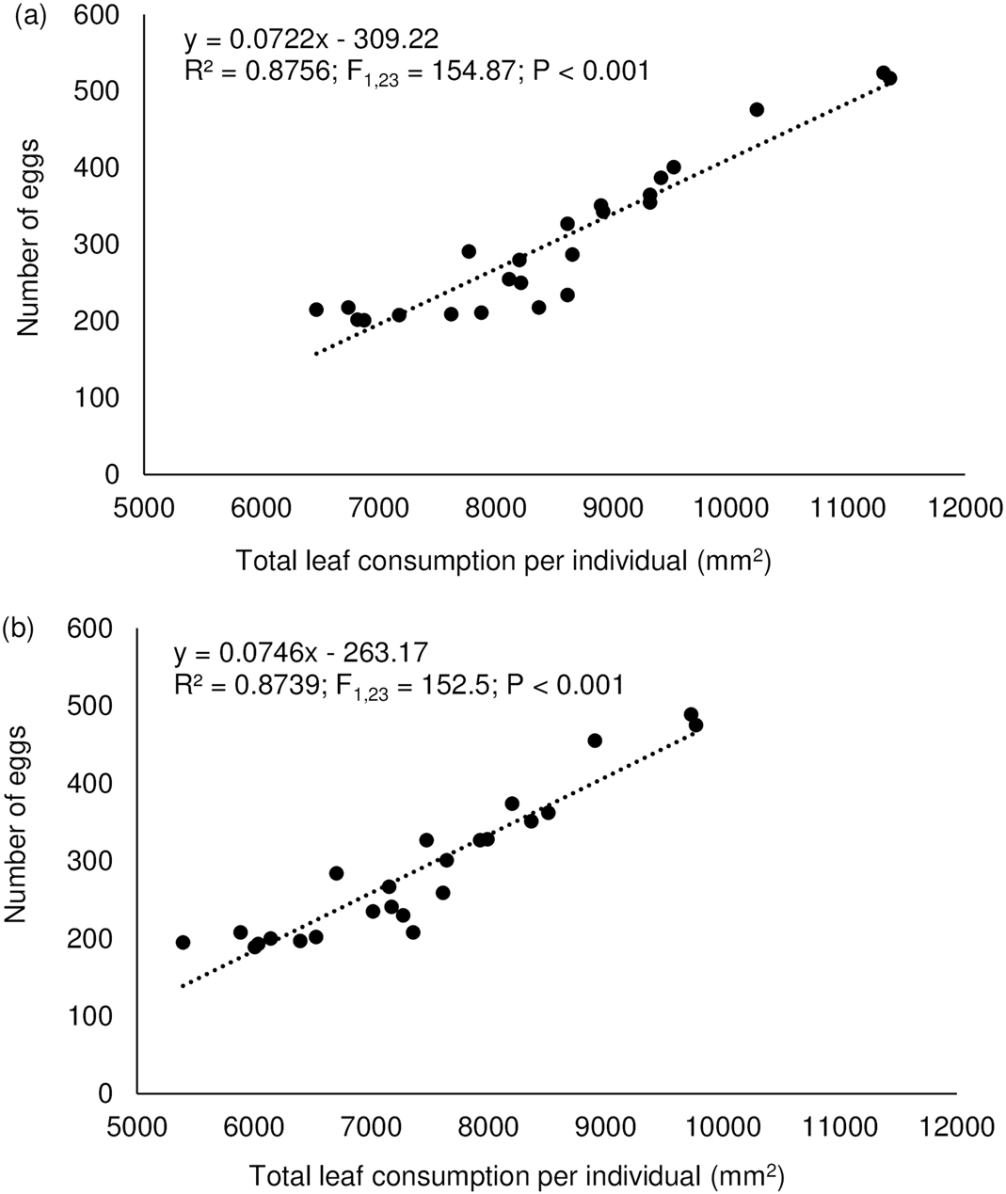
Relationship between number of eggs and total leaf consumption (mg) by the larvae of *Pareuchaetes pseudoinsulata* fed on shaded (**a**) and full-sun (**b**) leaves. Feeding was from first instar until pupation.

Irrespective of foliage types, linear regression analysis showed a significant positive relationship between the amounts of leaf tissue consumed and the resulting female pupal mass (Fig. 5a,b). A significant positive relationship between the amounts of leaf tissue consumed and the resulting number of eggs laid by females (reared on either foliage type) was also evident (Fig. 6a,b).

### Leaf characteristics

Light intensity in *C. odorata* growing habitats significantly influenced all measured leaf characteristics except phosphorus content (Table 3). Foliar nitrogen was higher in *C. odorata* plants growing in shaded environment, while carbon content was greater in full-sun leaves. However, phosphorus content of leaves was not significantly influenced by light intensity. The SLW (leaf toughness) of plants growing in full-sun was 46% greater than those of shaded plants whereas, leaf water content was significantly higher in plants growing in the shaded habitat compared with full-sun plants. Finally, total non-structural carbohydrate in leaves was 41% higher in full-sun plants compared with plants in the shaded habitat.

**Table 3:** Characteristics of leaves from *Chromolaena odorata* plants growing under full-sun or shaded habitats.

Characteristic	Full sun	Shade	*F*-value	DF	*P*-value
Nitrogen (% dry mass)	3.715 ± 0.051	4.846 ± 0.108	215.621	1, 19	**0.0001**
Non-structural carbohydrate (% dry mass)	5.374 ± 0.097	3.171 ± 0.109	227.31	1,19	**0.0001**
Phosphorus (% dry mass)	0.562 ± 0.019	0.504 ± 0.244	1.039	1, 19	0.086
Leaf water content (%)	72.472 ± 0.417	77.382 ± 0.397	44.268	1, 19	**0.0001**
Specific leaf weight (mg/mm^2^) (= leaf toughness)	0.057 ± 0.001	0.032 ± 0.001	135.218	1, 199	**0.0001**

Differences in means between habitat conditions (full-sun and shade) were compared using a one-way analysis of variance.

Statistically significant values are indicated in bold.

*DF* degrees of freedom.

### Discussion

In the present study, larvae of *P. pseudoinsulata* were more abundant on and caused greater damage to shaded leaves of *C. odorata* plants in the field compared to leaves of plants growing in full-sun. The results of this study suggest that this biological control agent may be more efficient against *C. odorata* in shaded habitats. A number of studies have reported that shading enhances the performance of weed biological control agents. For example, Lockett et al.^38^ recorded significant herbivory by a leaf-feeding geometrid, *Chiasmiaassimilis* (Warren) (Lepidoptera: Geometridae), on shaded *Acacianilotica* subsp. *indica* (Bentham) Brenan (Fabaceae). Beyond recommending the release of this biocontrol agent on *C. odorata* plants in shaded environments, biocontrol practitioners should prioritise studies on the impact of biocontrol agents under a variety of environmental conditions, as this can aid our understanding of potential outcomes of weed biocontrol programs. The preferential and greater herbivory of shaded *C. odorata* leaves by this moth might represent a strategy to avoid generalist predators such as birds. For example, in some plant species, it has been shown that some avian predators are unable to use cues of herbivore presence (emitted volatiles and/or changes in light reflectance of the leaves) to locate herbivorous insects in shaded conditions, compared to sunnier conditions where they are efficient at using such cues^39^. The high field abundance of larvae between June and September suggest that *P. pseudoinsulata* would perform better in rainy season.

In the laboratory experiment, the larvae of the moth consumed greater amounts of leaf material from shaded host plants. However, the greater consumption of shaded leaves by caterpillars did not influence the reproductive performance of *P. pseudoinsulata*. Female pupal mass, number of eggs laid, female longevity, mating success, duration of egg laying, pre-oviposition period, and egg hatchability were similar on both foliage types. The findings of this study further suggest that adults that fed on full-sun or shaded foliage as larvae would perform equally in terms of reproduction potential. Similar to the findings of Uyi et al.^40^ who investigated aspects of the biology of a related species (*P. insulata* [Walker]), total leaf consumption by *P. pseudoinsulata* on either foliage type was shown to be positively correlated with female pupal mass and number of eggs laid by females. Food quality/quantity is considered a good determinant of fecundity in herbivorous insects^41^—especially in capital breeders (i.e. species with adults that do not feed or ingest little food).

Most of the differences in leaf traits between plants grown under shade and under full-sun reported here are consistent with the predictions of the carbon-nutrient balance (CNB) hypothesis. This study showed that shaded leaves had lower leaf toughness, lower non-structural carbohydrate content, higher water content, and higher concentrations of foliar nitrogen than leaves from plants growing in full-sun. Shaded leaves also had higher abundance of *P. pseudoinsulata* and experienced increased leaf consumption in the field and in the laboratory. All these differences match expectations from the CNB hypothesis, which suggests that shading increases leaf palatability to herbivores^12–14^. However, the equal reproductive performance of moths fed on full-sun and shaded foliage is inconsistent with the CNB hypothesis and does not appear to extinguish the debates surrounding the hypothesis in terms of herbivore performance. Although, the CNB hypothesis predicts that leaves from plants in shaded environments are more suitable sources of food for insect herbivores and that herbivorous insects would perform better on such leaves, empirical evidence often suggests variable patterns^3–5,8,9,11,42^.

The finding that larvae of *P. pseudoinsulata* consumed significantly more shaded leaves but displayed equal reproductive performance metrics on both foliage types opens an interesting question about how insect herbivores respond to sunlight-mediated changes in the leaf characteristics of their host plants. The reduced leaf toughness, increased water and nitrogen contents, and the reduced NSC content in shaded leaves might be responsible for the increased leaf consumption by the larvae of *P. pseudoinsulata*. Sunlight-mediated variability in leaf toughness in plants remains central to explaining herbivory levels^1^ and insect performance metrics^8,11^, despite some reservations^3,9^. Generally, tougher (full-sun) leaves are less nutritious source of food and therefore herbivores consume less of, and perform poorly on, such leaves^1,4,8,11^. There are, however, convincing examples where the reverse is the case^3^. It is possible that the greater leaf toughness in the full-sun habitat contributes to the significantly reduced leaf consumption in this study. The increased water and nitrogen contents in shaded leaves might also have contributed to the increased leaf consumption in *P. pseudoinsulata*, as increased leaf water content and foliar nitrogen are often positively linked to leaf consumption or herbivory levels in several plant species^1,8^.

The increased consumption of the nitrogen-rich shaded leaves (compared to NSC-rich full-sun leaves) may be due to the reduced NSC in shaded leaves. Where foliar nitrogen and carbohydrate contents are imbalanced relative to requirements, insects will consume greater amounts of food to increase their intake of deficient nutrients^43,44^. Similar to the findings of this study, caterpillars feeding on high foliar nitrogen or high protein diets (e.g. shaded leaves), will consume large amounts of such protein rich diets to acquire carbohydrates that are in deficit^45^, thus redressing the carbohydrate deficit. Although this explanation may explain the greater intake of nitrogen-rich (or carbohydrate-poor) shaded leaves, it must be recognised that protein and carbohydrate are not often acquired in the ratio they are ingested^46^. At this point I consider sunlight mediated changes in leaf chemistry (increased nitrogen and reduced carbohydrates in shaded leaves), physiology (higher water content in shaded leaves) and defence (reduce toughness in shaded leaves) in the host plant as the most plausible explanation for the greater larval abundance on shaded plants, and increased consumption of shaded leaves by the larvae of *P. pseudoinsulata*. However, further comments are needed on the equal performance of the larvae on both foliage types in the laboratory.

The lack of significant differences in number of eggs laid and other reproductive performance metrics of the moth between full-sun and shaded foliage in this study suggests that foliar nitrogen content of leaves in full-sun plants (3.72%) satisfies the foliar nitrogen requirements of *P. pseudoinsulata*. A potential compensatory feeding response of *P. pseudoinsulata* to redress a carbohydrate deficit (relative to foliar nitrogen) may explain the lack of differences in the reproductive metrics measured in this study. The equal performance of the insect on both foliage types, despite the high foliar nitrogen concentration in shaded leaves (4.85%), indicates that increased foliar nitrogen levels in shaded leaves might have imposed a constraint on herbivore physiology that led to the neutral nutrient-performance relationships as has been documented in other studies^47–49^.

There are multiple nutritional ecological hypotheses^1^ that may explain the observed leaf consumption and herbivore performance pattern (increased herbivore consumption of shaded leaves but equal reproductive performance on shaded and full-sun leaves). First, insect herbivores, especially capital breeders may be expected to consume significantly greater amounts of foliage when fed on lower food quality of their host plants out of necessity in order to maintain fitness. A “necessity consumption” hypothesis may arise if an herbivore continues to feed on a poor quality host until the required nutrients necessary for pupation or adult emergence are obtained from the food—assuming there is a minimum larval or pupal mass necessary to allow for successful pupation or emergence. Second, the consumption of nutritionally superior leaves by herbivorous insects may cause larvae to grow more rapidly, and faster growing larvae may consume greater amounts of leaf—thereby resulting in a positive “feedback consumption” hypothesis^1^. A third hypothesis that may explain the observed pattern is the “luxury consumption” hypothesis where some larvae of herbivorous insects may consume nutritionally superior leaves beyond their immediate or basic requirements in order to gain a fitness advantage at latter life stages. For example, larvae consuming greater amounts of nutritionally superior leaves may have heavier final body mass, and larger mass (at the last instars) may result in increased successful pupation and emergence rates. Based on the equal reproductive performance of *P. pseudoinsulata* on full-sun and shaded foliage, the possibility of luxury consumption of the less tough and nitrogen-rich shaded foliage can be ruled out. The observed leaf consumption and herbivore performance pattern appears to be the result of feedback consumption of nitrogen-rich shaded foliage. The increased leaf consumption of shaded leaves in this study, and the faster development time as well as the higher growth rate of this moth on shaded leaves in a previous study^9^ seems to validate this feedback consumption hypothesis. The possibility of necessity consumption in this system cannot be jettisoned due to the following two reasons: (1) if higher foliar nitrogen concentration results in neutral nutrient-performance relationships (as observed in this study) or is associated with fitness costs^49–51^ in insects presumably because of elevated metabolic costs (related to catabolizing protein and excreting excess nitrogen)^48^, then the nitrogen-rich shaded leaves used in this study might impose some physiological constraints on the herbivore. The insect might have to frequently excrete excess nitrogen in its diet and the situation might warrant greater consumption of leaves until the required nutrients necessary for pupation or adult emergence are obtained from the food. (2) Another reason for a necessity consumption in this system is that the increased water in shaded leaves may have diluted the high nitrogen levels to such an extent that *P. pseudoinsulata* would have to consume greater amounts of the diluted shaded leaves until the required nutrients necessary for pupation or adult emergence are obtained from the food.

This current study suggests that larvae of *P. pseudoinsulata* will cause greater defoliation in *C. odorata* plants growing in shaded environments. The role of factors such as plant’s secondary chemicals, seasonality, climate (temperature and rainfall) and natural enemies in the biological control of *C. odorata* needs to be investigated. This study shows for the first time that an arctiine moth preferred a shaded habitat in the field and exhibited a feedback and/or necessity consumption pattern when fed on nitrogen and water rich shaded leaves of *C. odorata* plants. However, increased consumption of the nutritious shaded foliage did not result in improved herbivore reproductive performance. Whether or not these findings extend to other species is unknown and requires further studies on other systems. These data are crucial to advancing our understanding of the evolutionary and ecological factors driving insect herbivores response to light-mediated changes in leaf characteristics of their host plant species in nature.

## Data Availability

All relevant data are within the paper.
